# Endocrine therapy resistance of breast cancer: Important role of G protein-coupled estrogen receptor (GPER) and new therapeutic strategies

**DOI:** 10.1016/j.gendis.2025.101716

**Published:** 2025-06-10

**Authors:** Tenghua Yu, Chongwu He, Hui Zhang, Yi Zhu, Annie Wang, Xiaoqiang Zeng, Yanxiao Huang, Jiamin Zhong, Xingye Wu, Yi Shu, Guowei Shen, Chao Yu, Ke Zhou, Usman Zeb, Rebeka Dejenie, Yan Peng, Rex C. Haydon, Hue H. Luu, Russell R. Reid, Tong-Chuan He, Jiaming Fan, Jingjing Li

**Affiliations:** a Department of Breast Surgery, Jiangxi Cancer Hospital & Institute, The Second Affiliated Hospital of Nanchang Medical College, Jiangxi Key Laboratory of Oncology (2024SSY06041), Jiangxi Clinical Research Center for Cancer, JXHC Key Laboratory of Tumor Microenvironment and Immunoregulation, Jiangxi Key Laboratory of Tumor Metastasis of Jiangxi Health Commission, Nanchang, Jiangxi 330029, China; b Molecular Oncology Laboratory, Department of Orthopaedic Surgery and Rehabilitation Medicine, The University of Chicago Medical Center, Chicago, IL 60637, USA; c Department of Breast, Chongqing Hospital of Traditional Chinese Medicine, Chongqing 400021, China; d Ministry of Education Key Laboratory of Diagnostic Medicine, and Department of Clinical Biochemistry, School of Laboratory Diagnostic Medicine, Chongqing Medical University, Chongqing 400016, China; e Department of Gastrointestinal Surgery, The First Affiliated Hospital of Chongqing Medical University, Chongqing 400016, China; f Stem Cell Biology and Therapy Laboratory of the Pediatric Research Institute, The National Clinical Research Center for Child Health and Disorders, and Ministry of Education Key Laboratory of Child Development and Disorders, The Children's Hospital of Chongqing Medical University, Chongqing 400016, China; g Department of Orthopaedic Surgery, BenQ Medical Center, The Affiliated BenQ Hospital of Nanjing Medical University, Nanjing, Jiangsu 210019, China; h Department of Orthopaedic Surgery and Cardiology, The Affiliated University-Town Hospital, Chongqing Medical University, Chongqing 401331, China; i Department of Joint and Sports Medicine, Ningbo Medical Center Li Hui Li Hospital, Ningbo, Jiangsu 315040, China; j Institute of Biotechnology and Genetic Engineering, The University of Agriculture, Peshawar 25130, Pakistan; k School of Medicine, University of California Davis, Sacramento, CA 95817, USA; l Laboratory of Craniofacial Biology and Development, Section of Plastic and Reconstructive Surgery, Department of Surgery, The University of Chicago Medical Center, Chicago, IL 60637, USA; m Department of Oncology, The Affiliated Hospital, School of Clinical Medicine, Shandong Second Medical University, Weifang, Shandong 261053, China

**Keywords:** Breast cancer, Endocrine therapy, G protein-coupled estrogen receptor, Hormone receptor, Resistant mechanism, Therapeutic strategy

## Abstract

Breast cancer is the most prevalent malignancy that affects women worldwide, with approximately 70% of cases classified as hormone receptor-positive (HR^+^). Endocrine therapy is one of the principal treatment modalities for this patient cohort. However, a considerable proportion of tumors acquire resistance to endocrine therapeutics, resulting in reduced effectiveness as the disease progresses, but the underlying mechanisms are not fully characterized. The G protein-coupled estrogen receptor (GPER), a component of the G protein-coupled receptor family, is hypothesized to mediate estrogenic effects independently of conventional estrogen receptors. In recent years, our research group and others have demonstrated that GPER plays a crucial role in facilitating the clinical progression of HR^+^ breast cancer and significantly contributes to endocrine resistance. In this review, we summarize the diverse mechanisms through which GPER mediates endocrine resistance, encompassing somatic alterations, epigenetic and non-genetic variations, and modifications within the tumor microenvironment. Furthermore, we discuss GPER as a potential therapeutic target for overcoming endocrine resistance of HR^+^ breast cancer in future clinical applications.

## Introduction

Breast cancer (BC) is a frequently diagnosed malignancy, accounting for 23.8% of new incidences and 15.4% of mortality among cancer cases in women worldwide.^1^ Despite substantial advancements in therapeutic approaches to enhance survival in recent years, BC remains the foremost cause of cancer-related death in women.^1^ Several molecular mechanisms implicated in clinical drug resistance facilitate tumor progression and result in eventual relapse. Once the tumor metastasizes, the five-year survival rate for advanced BC patients is less than 25%.^2^ Thus, understanding the underlying intricacy of drug resistance and developing novel therapeutic strategies are essential for achieving better treatment outcomes and improving the dismal prognosis in these drug-resistant BC patients.

## BC classification and treatments

Currently, BC can be classified into diverse subtypes based on the presence of several clinical indicators: estrogen receptor (ER), progesterone receptor (PR), human epidermal growth factor receptor 2 (HER2), and Ki-67 index.^3^ Distinct combinations of these indicators define at least four primary subtypes of BC,^4^^,^^5^ which significantly contribute to individualized precision therapy (Table 1). Luminal A type: This subtype is positive for ER and/or PR (ER^+^ and/or PR^+^) and negative for HER2 (HER2^−^), has a low Ki-67 index, and makes up approximately 40% of all BC cases. The tumors are typically well-differentiated, exhibit slow growth, and tend to have the most favorable prognosis, with treatment typically involving anti-estrogen therapy.^3^^,^^6^^,^^7^ Luminal B type: Accounting for roughly 20%–30% of all BC cases, this subtype is ER^+^ and/or PR^+^, either HER2-positive (HER2^+^) or HER2^−^, and demonstrates a high level of Ki-67. The malignant biological behavior of luminal B BC is marginally higher than that of luminal A BC, and its prognosis is also slightly poorer.^3^^,^^6^^,^^7^ Pure HER2^+^ type: This subtype constitutes 10%–15% of all BC cases and is characterized by the absence of ER and PR and the overexpression of HER2. These tumors grow more rapidly than those of luminal tumors and generally have a poorer prognosis. However, they can be successfully treated by a combination therapy of chemotherapy and HER2-targeted therapy such as trastuzumab, pertuzumab, trastuzumab emtansine, and neratinib.^8^, ^9^, ^10^ Triple-negative type: Accounting for 15%–20% of all BC cases and characterized as negative for ER (ER^−^), PR (PR^−^), and HER2 (HER2^−^), the tumors are usually poorly differentiated and behave more aggressively than other BC types, making it the subtype with the poorest prognosis. Chemotherapy is usually the main treatment in the clinic, and combination with immunotherapy has also achieved significant breakthroughs in recent years.^6^^,^^7^^,^^11^

**Table 1:** Properties of the main clinical BC subtypes. BC can be classified based on the expression of certain receptors and Ki-67, which are associated with the proliferation and prognosis of BC, as well as the indicated clinical treatment approaches. BC, breast cancer.

BC subtypes	Luminal A	Luminal B	HER2 over-expression	Triple-negative
Proportion	40%	20%–30%	10%–15%	15%–20%
ER	+	+	–	–
PR	+/−	+/−	–	–
HER2	–	+/−	+	–
Ki-67	Low	Intermediate	High	High
Prognosis	Good	Intermediate	Intermediate	Poor
Treatment	Endocrine therapy	Endocrine therapy and HER2-targeted therapy (if HER2^+^)	Chemotherapy and HER2-targeted therapy	Chemotherapy and immunotherapy (if needed)

However, the above commonly employed clinical classifications are inadequate to fully deal with issues of tumor heterogeneity and drug resistance. The advent of RNA-based molecular profiling is conducted to improve our understanding of BC heterogeneity and impact BC stratification and treatment options. Because of pioneering microarray expression profiling studies, five primary intrinsic molecular subtypes, namely luminal A, luminal B, HER2-enriched, basal-like, and claudin-low, exhibit distinctive biological, prognostic, and clinical characteristics.^12^^,^^13^ Recently, Jiang et al made a considerable advancement in this field by utilizing next-generation DNA and RNA sequencing combined with metabolomics and proteomics. Luminal BC can be further identified into the following four molecular subtypes: canonical luminal, immunogenic, proliferative, and receptor tyrosine kinase (RTK)-driven.^14^ HER2-overexpressing BC is composed of classical HER2, immunomodulatory, luminal-like, and basal/mesenchymal-like subtypes.^15^ Triple-negative BC is classified into four transcriptome-based subtypes: luminal androgen receptor, immunomodulatory, basal-like immune-suppressed, and mesenchymal-like.^16^^,^^17^ These distinct molecular subtypes lay the foundation for developing more precise treatment strategies based on individualized patient characteristics (Table 2).

**Table 2:** Precision molecular classification of diverse BC subtypes. Luminal, HER2 overexpression, and triple-negative BC can be further categorized based on their genetic and metabolic characteristics, which are related to the prognosis and precision treatment strategies for BC patients. BC, breast cancer; HER2, human epidermal growth factor receptor 2.

BC subtypes	Luminal	HER2 over-expression	Triple-negative
Classificaton and proportion	1) Canonical luminal (24.5%) 2) Immunogenic (25.4%) 3) Proliferative (33.6%) 4) RTK-driven (16.5%)	1) Classical HER2 (28.3%) 2) Immunomodulatory (20%) 3) Luminal-like (30.6%) 4) Basal/mesenchymal-like (21.1%)	1) Luminal androgen receptor (23%) 2) Immunomodulatory (24%) 3) Basal-like immune-suppressed (39%) 4) Mesenchymal-like (15%)
Genetic and metabolic characteristics	1) Enriched of PAM50 luminal A, high *PlK3CA*^mut^ and low *TP53*^mut^ 2) Low chromosomal instability, high *TP53*^mut^ and enriched of immune cells 3) High chromosomal instability, high metabolism abnormal, enriched of PAM50 luminal B, activation of cell cycle, and high homologous recombination deficiency (HRD) score 4) RTK pathway activation	1) High *ERBB2* activation 2) Immune-activated microenvironment 3) ER signaling activation 4) RTK pathway activation	1) High prevalence in Asians, PI3K/AKT and *ERBB2* activation and low chromosomal instability 2) High tumor infiltrating lymphocytes (TILs) infiltration 3) Enrichment of the HRD mutation signature and high chromosomal instability 4) Characteristics of stem-like cells and JAK/STAT3 activation
Possible prognosis	1) Good 2) Intermediate 3) Intermediate 4) Poor	1) Intermediate 2) Good 3) Poor 4) Poor	1) Intermediate 2) Good 3) Intermediate 4) Poor
Potential treatment	1) Endocrine therapy and PI3K inhibitors 2) Immune checkpoint inhibitors 3) CDK4/6 inhibitors and Poly-ADP-ribose polymerase (PARP) inhibitors 4) RTK inhibitors	1) Standard HER2-targeted therapy 2) De-escalated therapy and immunotherapy 3) Standard HER2-targeted therapy and CDK4/6 inhibitors 4) Standard HER2-targeted therapy and RTK inhibitors	1) Endocrine therapy, targeting ERBB2 and CDK4/6 inhibitors 2) Immune checkpoint inhibitors 3) Low HRD score: Escalated chemotherapy, intensive monitoring; high HRD score: Platinum drugs or PARP inhibitors 4) STAT3 inhibitors

**Figure 1:**
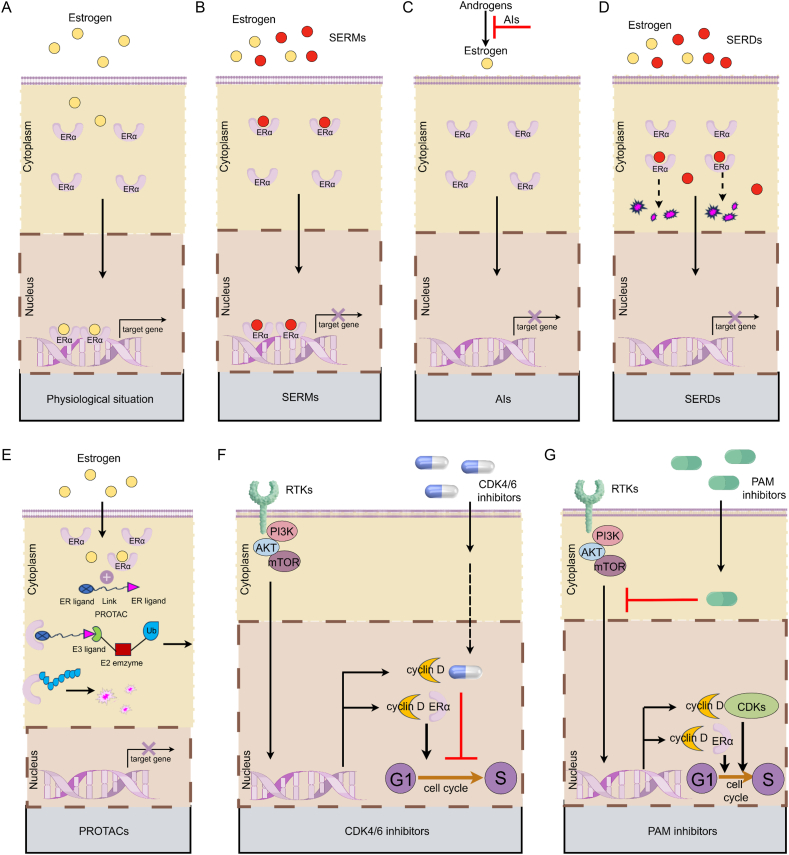
The mechanism of estrogenic action through nuclear ERα in physiological situations and of common clinical endocrine therapies. **(A)** In a physiological situation, when binding to synthesized estrogen, the ERα dimerizes and translocates to the nucleus, binding co-activators to form a transcriptionally active ER complex and subsequently inducing the expression of downstream target genes. **(B)** SERMs, such as tamoxifen, toremifene, and raloxifene, competitively inhibit the binding of estrogen to ERα. **(C)** AIs, such as letrozole, anastrozole, and exemestane, prevent estrogen production by inhibiting the aromatization of androgens to estrogens. **(D)** SERDs, such as fulvestrant, are regarded as pure ERα antagonists. The inhibitory effect of SERDs is attributed to the reduced ability of SERD-bound ERα to translocate to the nucleus. SERD-bound ERα undergoes degradation because of impaired mobility. **(E)** PROTACs are heterobifunctional molecules that consist of a ligand for ERα and another ligand that serves as a substrate for the E3 ubiquitin ligase complex. Once binding to ERα, PROTACs recruit the E3 ubiquitin ligase complex, which polyubiquitinates ERα and marks it for proteasomal degradation. **(F)** CDK4/6 inhibitors, such as palbociclib, ribociclib, abemaciclib, and dalpiciclib, target the CDK4/6 complex, hindering cancer cells from entering the G1 checkpoint to the S phase of the cell cycle. **(G)** PAM inhibitors, such as alpelisib, capivasertib, everolimus, and inavolisib, target the inhibition of key molecules in the PAM signaling pathways to achieve the purpose of inhibiting the cell cycle and growth of BC cells. ERα, estrogen receptor alpha; SERMs, selective ER modulators; AIs, aromatase inhibitors; SERDs, selective ER downregulators; PROTACs, proteolysis targeting chimeras; CDK4/6, cyclin-dependent kinase 4/6; PAM, PI3K/AKT/mTOR.

Among these subtypes, the hormone receptor-positive (HR^+^) subtype, including luminal A and luminal B, is the most prevalent subtype and accounts for about 70% of all BC malignancies,^18^ thereby representing a large proportion of patients in the clinic. Endocrine therapies have significantly reduced the recurrence and mortality of HR^+^ BC.^19^ A variety of endocrine treatment drugs (Fig. 1), such as selective ER modulators (SERMs), aromatase inhibitors (AIs), and selective ER downregulators (SERDs), have been approved for adjuvant or rescue treatment in HR^+^ BC.^20^ Furthermore, recent efforts in drug development of next-generation ER-targeted agents, including oral SERDs and proteolysis targeting chimeras (PROTACs)-ER degraders, also offer another promising treatment option.^21^ The addition of cyclin-dependent kinase 4/6 (CDK4/6) inhibitors (*e.g.*, palbociclib, ribociclib, abemaciclib, and dalpiciclib) to antiestrogen drugs has notably prolonged the progression-free survival in patients with HR^+^ metastatic BC (mBC).^22^, ^23^, ^24^, ^25^ More importantly, there is a statistically significant benefit in terms of invasive disease-free survival from the addition of abemaciclib for 2 years or ribociclib for 3 years in combination with adjuvant hormone therapy in HR^+^ early BC with a moderate- or high-recurrence risk.^26^^,^^27^ Although numerous advancements have taken place in the field of endocrine therapy, the development of endocrine resistance remains a considerable challenge, which may lead to a poor prognosis for HR^+^ BC in certain patients. Endocrine resistance may involve complex mechanisms, such as somatic alterations, epigenetic variations, and changes in the tumor microenvironment.^28^ The core factors that trigger these resistant mechanisms still need to be elucidated.

## Genomic actions of classical ERs

The so-called classical ERs, namely ERα and ERβ, are nuclear ERs that mainly function as ligand-activated transcription factors.^29^^,^^30^ Once estrogen binds to ERα, it enables the receptor to dimerize, translocate to the nucleus, and interact with transcriptional co-activators (such as activator protein 1/AP-1 and specific protein 1/SP-1), or directly bind to estrogen-responsive elements within the promoters of target genes (such as MYC and cyclin D1).^31^ These genomic actions regulate the cell's oncogenic potential and increase cancer cell proliferation, differentiation, and the risks of DNA damage.^32^^,^^33^ Although targeting ERα signaling is widely utilized in clinical practice, alterations in ERα itself, including changes in expression level, estrogen receptor 1 (ESR1) mutation, or ERα signaling self-activation, frequently lead to endocrine resistance.^34^^,^^35^ Moreover, ERβ is another nuclear receptor homologous to ERα that is encoded by the estrogen receptor 2 (*ESR2*) gene and has a structure similar to that of ERα.^36^ It interacts with some ERα transcriptional co-regulators and shares similar ligands with ERα, but with different affinities. In contrast to ERα, activation of ERβ generally leads to the inhibition of proliferation, migration, and invasion in HR^+^ BC,^37^ and the ERβ-mediated anti-hormonal treatments can be beneficial for triple-negative BC therapy.^38^ Interestingly, although ERβ reduces AKT signaling with subsequent decreased effects on proliferation, survival, and improved tamoxifen sensitivity of BC cells,^39^ Meligova et al report that ERβ2 (an isoform of ERβ) desensitizes MCF-7 cells to the inhibitory effects of tamoxifen and fulvestrant, serving as a marker of endocrine resistance.^40^ Clinically, the dual roles of ERβ in breast tumors tend to correlate with significant differences in patient prognosis.^41^ Thus, further research is needed to better understand the potential factors that regulate the physiological role of classical ERs and their involvement in endocrine-resistant mechanisms.

## GPCR family and non-genomic actions of GPERs

G protein-coupled receptors (GPCRs) represent the largest family of transmembrane proteins and transmute a wide range of physical and chemical stimuli within the cell by coupling with intracellular heterotrimeric G proteins, thereby governing multiple downstream signaling pathways and transcriptional programs.^42^^,^^43^ The GPCR repertoire is expanded and diversified to meet the escalating need for intercellular communication in the evolution of multicellular organisms. Numerous efforts are being exerted to illuminate the complex circuitry of GPCR signaling at both intracellular and extracellular ligand levels,^44^, ^45^, ^46^, ^47^ which modulate multiple cellular functions integral to the features of cancer, such as cell growth, proliferation, migration, metabolism, death, and drug resistance.^48^ Even though GPCRs are not regarded as "genetic drivers" of cancer, their roles in tumor progression have been firmly established. Hence, GPCRs are considered excellent therapeutic targets for the following reasons^49^^,^^50^: i) GPCRs are typically highly expressed in pan-tumors; ii) The expression of GPCRs is independent of the tumor stage and grade; iii) GPCRs are not frequently mutated in cancer tumors (less than 1% of tumor samples), making them promising as stable targets for drug development. Nevertheless, there remain numerous challenges in translating these GPCRs as targets into clinical treatment. Tumors typically express over 150 distinct GPCRs, and nearly half display altered expression levels in the pathogenic stage.^49^ As such, it is still extremely difficult to identify precise and effective GPCR targets. Although hundreds of GPCR-targeting drugs have been approved or are under development for various diseases, no drugs have been approved yet for clinical use in BC patients. Therefore, during long-term endocrine resistance in HR^+^ BC, identifying a clinical target with both GPCR and ERα functions will facilitate our better exploration of the resistant mechanisms and present opportunities to develop more accurate drugs with greater efficacy and minimal adverse reactions.

**Figure 2:**
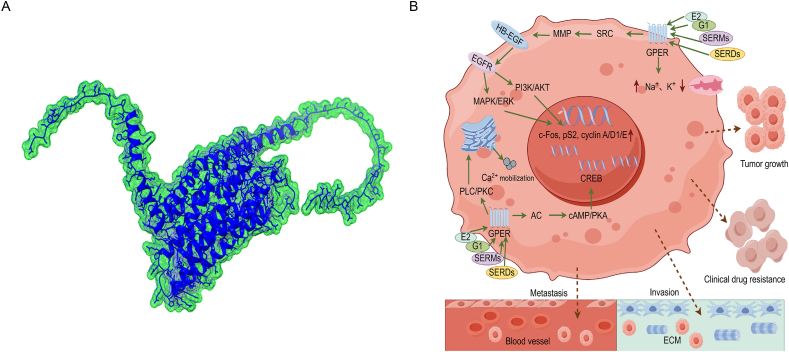
The predictive protein structure of GPER and its intracellular downstream non-genomic signaling pathways. **(A)** The AlphaFold protein structure of GPER from the public database (https://alphafold.com/entry/Q99527). **(B)** E2, the GPER-specific agonist G1, SERMs, and SERDs activate GPER, which is predominantly localized at the cell membrane or intracellularly at the endoplasmic reticulum. GPER activates several heterotrimeric G proteins, leading to the activation of SRC, which further induces the activation of MMPs, cleaves HB-EGF, and releases free HB-EGF. HB-EGF then transactivates the EGFR, which in turn activates the downstream pathways of MAPK/ERK and PI3K/AKT, inducing gene transcription (such as c-Fos, pS2, cyclin A, cyclin D1, and cyclin E) and regulating ion channels (such as Ca^2+^, Na^+^, and K^+^). Other G protein-activated multiple downstream cascades include the activation of AC and the production of cAMP, followed by the activation of the PKA/CREB axis. The mobilization of Ca^2+^ from intracellular stores activates the PLC/PKC axis. The above signalings co-regulate downstream gene expression and promote tumor growth, invasion, metastasis, and clinical drug resistance. GPER, G protein-coupled estrogen receptor; SERMs, selective ER modulators; SERDs, selective ER downregulators; MMPs, matrix metalloproteinases; HB-EGF, heparin-binding epidermal growth factor; MAPK, mitogen-activated protein kinase; ERK, extracellular signal-regulated kinase; PI3K, phosphoinositide 3-kinase; AKT, protein kinase B; AC, adenylate cyclase; cAMP, cyclic adenosine monophosphate; PKA, protein kinase A; CREB, cAMP-response element binding protein; PLC, phospholipase C; PKC, protein kinase C.

GPER, a GPCR family member consisting of 375 amino acid residues (Fig. 2A) and featuring the characteristic traits of GPCRs, was identified and cloned in 1997, which was formerly known as G protein-coupled receptor 30 (GPR30).^51^^,^^52^ Three physiological forms of endogenous estrogens, namely estrone, 17β-estradiol (E2), and estriol, are known to bind to GPER. Estrone and E2 function as agonists, while estriol acts as an antagonist, and GPER shows no binding to other steroids, such as testosterone, progesterone, aldosterone, and cortisol.^53^, ^54^, ^55^ Exogenous estrogens, like bisphenol A (BPA) and phytoestrogens, are mainly derived from diet and environment.^56^ Although GPER can be activated by E2, it presents an independent ER that is not homologous to nuclear ERα and ERβ and acts rapidly in a ligand-dependent manner (namely non-genomic effect), making GPER the third novel type of ER.^57^ Briefly, the main GPER signaling can be described as follows: The activation of GPER leads to the activation of Src family tyrosine kinases and matrix metalloproteinases (MMPs), and then promotes the release of heparin-binding epidermal growth factor (HB-EGF). HB-EGF transactivates EGFR, further activates the mitogen-activated protein kinase/extracellular signal-regulated kinase (MAPK/ERK) and phosphoinositide 3-kinase/protein kinase B (PI3K/AKT) pathways, then up-regulates the expression of the proto-oncogenes c-Fos, pS2, and cyclin A, cyclin D1, and cyclin E, and regulates ion channels, including calcium, sodium,^58^ and potassium.^59^ The activated GPER also mobilizes the calcium pool, causing a rapid increase in intracellular Ca^2+^, and stimulates adenylate cyclase as well as elevating the intracellular level of the second messenger cyclic adenosine monophosphate/protein kinase A (cAMP/PKA),^60^ and increases the involvement of the phospholipase C/protein kinase C (PLC/PKC). The aforementioned GPER signaling further influences other bypass signaling and downstream gene expression, and regulates tumor growth, invasion, metastasis, and clinical drug resistance (Fig. 2B).

Interestingly, clinical findings suggest that GPER and ERα are independently expressed in BC tissues. 19% of BC patients present HR^+^/GPER^−^, and 43% of all patients demonstrate HR^+^/GPER^+^, indicating that approximately 70% of tumor tissues in HR ^+^ patients express GPER.^61^ Furthermore, studies have also disclosed that GPER plays an estrogen-mediated pro-cancer role by disrupting the homeostasis of breast tumor glands, promoting cancer progression, and leading to deteriorating prognoses,^62^ especially as the mechanisms of endocrine resistance may have numerous cross-talks with GPER or its related signaling. In this report, we conducted a review of the detailed mechanisms underlying the GPER-mediated regulation of endocrine resistance in HR^+^ BC. Moreover, our review identified possible therapeutic approaches targeting GPER that may alleviate endocrine resistance or enhance the treatment efficacy of HR^+^ BC in the future.

## Clinical findings between GPER and endocrine therapies in HR^+^ BC

Different endocrine therapeutics protect HR^+^ BC patients through diverse mechanisms (Fig. 1B–G). For example, SERMs (*e.g.*, tamoxifen, toremifene, raloxifene, and lasofoxifene) compete with estrogen for binding to ER and are mainly utilized in pre-menopausal BC. AIs (*e.g.*, anastrazole, letrozole, and exemestane) lower systemic estrogen levels in post-menopausal BC by preventing the conversion of androgens to estrogens. Intramuscular or oral SERDs (*e.g.*, fulvestrant, elacestrant, camizestrant, and giredestrant) are believed to operate primarily by inducing ER protein degradation or blocking ER transcriptional activity.^63^ PROTACs-ER (vepdegestrant, ARV-471) facilitates the interaction between ER and the ER3 ligase complex, leading to ER ubiquitination for proteasomal degradation.^64^ CDK4/6 inhibitors target the CDK4/6 complex, thereby promoting the activation of retinoblastoma protein, which hinders cancer cells from entering the G1 checkpoint to the S phase of the cell cycle.^65^ PI3K/AKT/mTOR (PAM) inhibitors in combination with endocrine therapies have also been approved for the second- or posterior-line treatment of HR^+^ mBC.^66^ These relatively mature endocrine drugs are extensively used in clinical practice.

## GPER and SERMs

**Figure 3:**
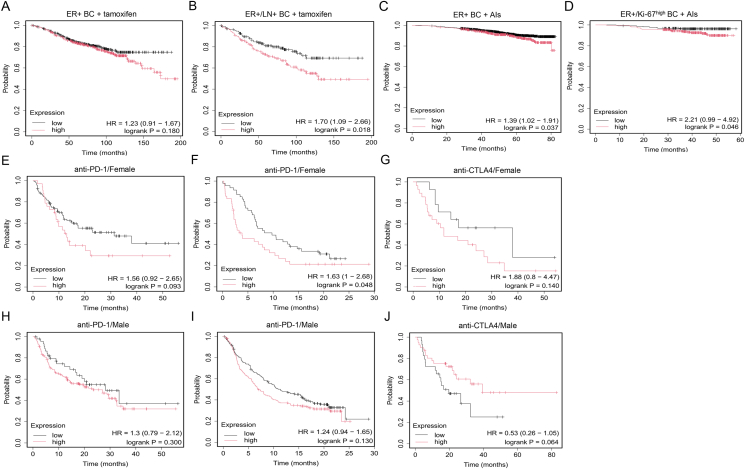
The association of GPER expression with clinicopathological characteristics in various cancer patients. **(A, B)** The connection between GPER expression and OS rate in ER^+^ BC and high-risk LN^+^/ER^+^ BC patients undergoing tamoxifen treatment. **(C, D)** The relationship between GPER expression and RFS rate in ER^+^ BC and high-risk Ki-67^high^ ER^+^ BC patients receiving AI treatment. Highly GPER expression indicates a poor prognosis in clinical high-risk ER^+^ BC patients undergoing endocrine treatments (B, D). **(E**–**J)** The association between GPER expression and RFS rate in female or male patients with different solid tumors (melanoma, lung cancer, liver cancer, bladder cancer, *etc*.) under the administration of diverse immunotherapies (*e.g.*, anti-PD-1, anti-PD-L1, and anti-CTLA4). A sex-specific phenomenon is noted where elevated GPER expression tends to result in a poor immunotherapy response in female patients (E–G), which significantly differs from that of male patients (H–J). GPER, G protein-coupled estrogen receptor; OS, overall survival; ER^+^, estrogen receptor-positive; BC, breast cancer; LN^+^, lymph node-positive; RFS, relapse-free survival; AI, aromatase inhibitor; PD-1, programmed cell death-1; PD-L1, programmed cell death-ligand 1; CTLA4, cytotoxic T lymphocyte-associated antigen 4.

Strong evidence has shown a distinct clinical correlation between GPER and tamoxifen resistance. Long-term survival follow-up data show that GPER expression is associated with a decreased response rate to tamoxifen therapy in BC patients,^67^, ^68^, ^69^, ^70^ and this finding was supported by a public dataset^71^ for the discovery and validation of survival-associated GPER genes in high-risk lymph node-positive ER^+^ BC, who receive tamoxifen treatment (Fig. 3A, B). Our team further identified that GPER was an initiator of tamoxifen resistance in hormone-dependent BC, and long-term endocrine treatment facilitated the translocation of cell membrane GPER to enhance its downstream resistant signaling.^72^, ^73^, ^74^ Similar outcomes were also observed where continuous exposure of tamoxifen fed back to up-regulate GPER and increase tumor progression.^75^, ^76^, ^77^, ^78^, ^79^ Interestingly, GPER-induced tamoxifen resistance leads to secondary decreased chemosensitivity by regulating the expression and localization of ATP binding cassette subfamily G member 2 (ABCG2),^74^ suggesting that GPER plays a crucial role in triggering multiple drug resistance in BC. Furthermore, apart from tamoxifen, GPER can also be activated by other SERM drugs like raloxifene and may influence raloxifene sensitivity *in vitro*.^80^^,^^81^ However, due to the relatively limited universality of clinical applications, no clinical data regarding GPER and other SERMs resistance have been reported.

## GPER and AIs

Currently, there is no direct clinical prognostic evidence suggesting that GPER participates in resistance to AIs. Nevertheless, bioinformatics data from public datasets revealed that GPER might be implicated in this event. High-risk Ki-67^high^ ER^+^ BC patients who are treated with AIs and have high GPER expression showed a significantly reduced relapse-free survival time compared with the population with low GPER expression (Fig. 3C, D). Moreover, treatment with AI drugs, including anastrozole, letrozole, and exemestane, caused considerable expression changes of GPER in various disease models *in vitro* and to a certain extent *in vivo*,^82^, ^83^, ^84^, ^85^, ^86^, ^87^, ^88^ suggesting a potential association between GPER and AI resistance. Thus, long-term follow-up data should be further explored.

The serum level of estrogen is of great significance for AI sensitivity in HR^+^ BC, and ovarian function suppression is widely employed in premenopausal patients. Conversely, in postmenopausal patients, the aromatase gene cytochrome P450 family 19 subfamily A member 1 (*CYP19A1*) may be altered in AI-resistant tumors. *CYP19A1* amplification was reported in 21.5% of AI-treated HR^+^ mBC and led to enhanced aromatase activity, inducing non-steroidal AI resistance.^89^ Interestingly, tamoxifen up-regulates *CYP19A1* expression through GPER not only in tumor cells themselves but also in cancer-associated fibroblasts (CAFs) from the tumor microenvironment.^90^^,^^91^ This data implies that AI therapy alone may not offer sufficient sensitivity once the HR^+^/GPER^+^ breast tumor develops tamoxifen resistance. Therefore, the inclusion of the GPER-induced aromatase gene in targeted sequencing panels may identify a subset of AI-resistant tumors that would be strong candidates for other treatment methods, such as the combination of SERDs with CDK4/6 inhibitors or other target drugs.

## GPER and SERDs

SERDs are currently considered indispensable as either single or combined therapeutics for the treatment of ER^+^ mBC. Among them, fulvestrant is the first approved SERD and is administered intramuscularly. Recently, numerous oral SERDs such as elacestrant, camizestrant, giredestrant, and amcenestrant have been developed to overcome the limitation of intramuscular administration and enhance the bioavailability of SERDs in several phase II-III clinical trials.^92^^,^^93^ Currently, clinical data regarding GPER and prognosis in patients treated with SERDs are limited. Giessrigl et al discovered that fulvestrant causes resistance by modulating the expression of GPER and CDK6 through the involvement of methyltransferases, deacetylases, and the human SWItch/Sucrose nonfermentable chromatin remodeling complex in the HR^+^ BC cells.^94^ Combined with numerous previous studies, it has been found that fulvestrant acts as an agonist for GPER, initiating its downstream signaling to promote the malignant biological behavior of BC.^74^^,^^95^, ^96^, ^97^, ^98^, ^99^ Therefore, sufficient follow-up time is still necessary to explore the association between GPER and SERD sensitivity in the clinic.

## GPER and PROTACs

PROTACs are hetero-bifunctional small molecules that recruit an E3-ubiquiting ligase to the target protein and have the potential to degrade both ERs and GPER. Two ERs/GPER-targeting PROTACs (UI-EP001 and UI-EP002) have been designed, which effectively degrade ERα, ERβ, and GPER, inducing cytotoxicity and G2/M cell cycle arrest in MCF-7 (ERα^+^/ERβ^+^/GPER^+^) and SKBR3 (ERα^−^/ERβ^−^/GPER^+^) BC cells but not in MDA-MB-231 BC cells that do not express functional ERα/ERβ/GPER.^55^ However, the efficacy of monotherapy with PROTACs remains suboptimal, and combination therapy with CDK4/6 or PAM inhibitors may present a promising approach to enhance the treatment outcome.^100^ Therefore, we hypothesize that targeting GPER signaling in combination with PROTACs will be a significant research direction to overcome clinical endocrine resistance.

## GPER and CDK4/6 inhibitors

Endocrine therapy in combination with CDK4/6 inhibitors currently is the standard adjuvant therapy for moderate-to high-risk HR^+^ early BC and the first-line salvage therapy for HR^+^ mBC.^101^^,^^102^ Despite the considerable advancement of CDK4/6 inhibitors in the treatment of BC, tumor resistance to CDK4/6 inhibitors has been globally reported,^103^^,^^104^ and there is an urgent need to explore their specific mechanisms. Approximately 20% of patients with BC do not respond to CDK4/6 inhibitors,^105^ which indicates that a considerable number of HR^+^ BC might encounter this type of resistance. Intriguingly, the down-regulation of ERα along with the up-regulation of GPER was discovered in palbociclib-resistant ER^+^ BC cells, and palbociclib induces pro-inflammatory events via GPER signaling in CAFs, mutually contributing to the decreased sensitivity to CDK4/6 inhibitors.^106^ Furthermore, GPER mediates the increased expression of various cyclins,^107^^,^^108^ such as cyclin D1, cyclin E, and cyclin A, which may be further implicated in the resistance to CDK4/6 inhibitors. Considering the potential differences in the pharmacological mechanism of diverse CDK4/6 inhibitors, further exploration of the mechanism of GPER and resistance to various CDK4/6 inhibitors is expected to improve the prognosis of HR^+^ BC.

## GPER and PAM inhibitors

PAM inhibitors, such as the PI3K inhibitor alpelisib,^109^ the AKT inhibitor capivasertib,^110^ and the mTOR inhibitor everolimus,^111^ have been approved as clinical options following resistance to CDK4/6 inhibitors in HR^+^ mBC. Inavolisib, a highly selective inhibitor of the alpha isoform of the p110 catalytic subunit of the phosphatidylinositol 3-kinase complex (PIK3CA), was recently approved for PIK3CA-mutated patients with HR^+^ mBC,^112^ as its combination therapy with palbociclib and fulvestrant led to significantly longer progression-free survival compared with the placebo plus palbociclib and fulvestrant group. However, the role of PAM inhibitors in second-line or post-line therapy for mBC is still limited, as a considerable number of resistance events have been witnessed in the clinic. The precise mechanism still needs to be further explored. Additionally, the clinical application of PAM inhibitors depends on costly genetic testing, thereby restricting their use in the clinic. Intriguingly, GPER signaling shows significant crosstalk with the PAM pathways in various disease models, including BC,^113^, ^114^, ^115^, ^116^, ^117^, ^118^ suggesting that the combination of targeting GPER and PAM inhibitors might constitute an effective treatment option, which can be achieved using convenient and inexpensive immunohistochemical methods.

## Mechanisms underlying GPER-induced somatic alterations

**Figure 4:**
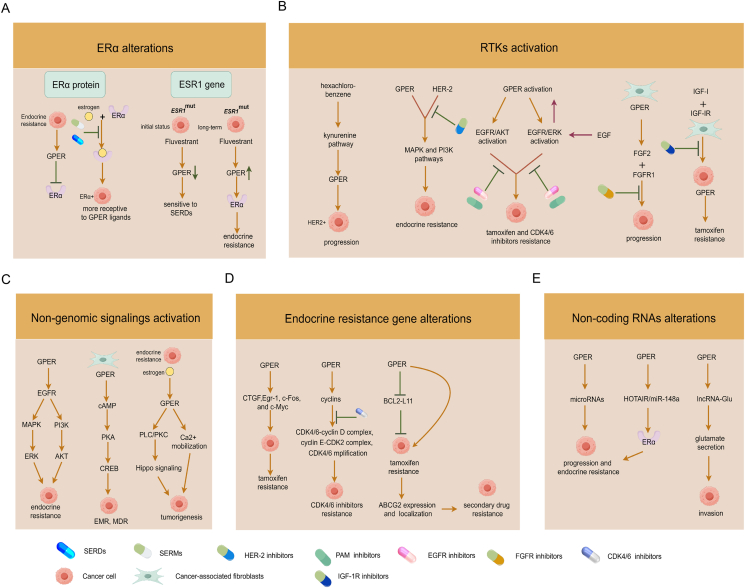
Mechanisms of GPER-induced somatic alterations. The roles of GPER in **(A)** ERα alterations, **(B)** RTK activation, **(C)** non-genomic signaling activation, **(D)** endocrine resistance gene alterations, and **(E)** ncRNA alterations are shown. GPER, G protein-coupled estrogen receptor; ERα, estrogen receptor alpha; RTK, receptor tyrosine kinase.

Large-scale DNA sequencing maps of HR^+^ mBC have clarified the significance of somatic alterations related to the poor response to endocrine therapy.^119^, ^120^, ^121^ These alterations encompass ERα alterations, activation of RTKs and non-genomic signaling, and alterations of endocrine resistance genes and non-coding RNAs (ncRNAs) (Fig. 4).

## GPER and ERα alterations

Our previous research revealed that negative correlations were observed between GPER and ERα and PR expression.^122^^,^^123^ In fact, during the long-term course of endocrine drug resistance in HR^+^ BC, down-regulation of ERα expression and up-regulation of GPER expression have been identified,^72^^,^^106^ suggesting a potential regulatory mechanism of GPER-induced ERα alterations. Furthermore, GPER co-expressed with ERα is detected in 36.6% of BC tissues.^124^ This co-expression is regulated by estrogen-ERα signaling in ERα^+^ BC cells, and estrogen-mediated activation of GPER makes the tumor cells more receptive to GPER ligands.^125^ Collectively, this data indicates that there is a complex mutual regulatory relationship between GPER and ERα.

Except for the expression of ERα itself, the occurrence of mutation in the estrogen receptor 1 (*ESR1*^mut^) gene has a well-defined mechanism, which occurs in approximately 30% of cases presenting endocrine therapy resistance.^126^^,^^127^ The influence of *ESR1*^mut^ on the activity of endocrine therapy may significantly lead to resistance to AIs, while generally maintaining variable sensitivity to SERDs.^126^^,^^128^ The possible reason might be that the initial treatment of fluvestrant may cause a certain down-regulation of GPER,^94^ making tumor cells temporarily sensitive to SERDs. However, after long-term stimulation by fluvestrant, GPER signaling is gradually activated and then regulates ERα function to evoke endocrine resistance. Moreover, the analysis of the Cancer Genome Atlas database suggested that *ESR1*^mut^ and GPER gene co-expression predicted a poor overall survival time and metastatic stage of BC patients.^118^ Therefore, continuous in-depth studies of GPER-induced ERα alterations will help us better understand the mechanisms of GPER-mediated endocrine resistance.

## GPER and RTKs activation

HER2 (*ERBB2*) gene amplification has long been regarded as a factor that reduces sensitivity to anti-estrogen treatment. The standard treatment for ER^+^/HER2^+^ BC involves a combination of anti-estrogens and HER2 inhibitors.^129^ Interestingly, GPER expression showed a strong positive correlation with HER2 overexpression, histological grade, large tumor size, and more metastatic disease.^123^^,^^130^ Exposure to environmentally relevant concentrations of hexachlorobenzene up-regulates the kynurenine pathway and GPER levels, contributing to the progression of HER2^+^ BC.^131^ This indicates that GPER^+^/HER2^+^ BC exhibits relatively higher malignant behavior. Both GPER and HER2 can mediate downstream signaling such as the MAPK and PI3K pathways, which significantly contribute to endocrine resistance. Furthermore, HER2-activating mutations are also identified in approximately 5% of endocrine-resistant mBC, causing these tumors to be not only resistant to estrogen deprivation and fulvestrant but also respond poorly to the HER2 tyrosine kinase inhibitor.^132^^,^^133^ Although the correlation between GPER and HER2-activating mutations remains unknown, we infer that simultaneous targeting of ER and HER2 might not be sufficient for the classical triple-positive (ER^+^/PR^+^/HER2^+^) BC. We need to pay more attention to the new triple-positive subtype (ER^+^/GPER^+^/HER2^+^) and investigate whether simultaneous targeting of ER, GPER, and HER2 may be better at overcoming endocrine resistance.

EGFR amplification is detected in 1.7% of endocrine-resistant mBC.^134^ EGFR mediates fulvestrant resistance, which can be reversed by combining EGFR or ERK inhibitors.^135^ A large number of studies have confirmed that GPER activation is accompanied by downstream EGFR/ERK or EGFR/AKT activation and further enhances tamoxifen and CDK4/6 inhibitor resistance.^73^^,^^74^^,^^91^^,^^97^^,^^106^^,^^136^, ^137^, ^138^, ^139^, ^140^, ^141^, ^142^, ^143^ Conversely, epidermal growth factor induces GPER expression through the EGFR/ERK transduction pathway, generating positive feedback for the activation of GPER signaling.^144^^,^^145^ Thus, precise blocking of the GPER/EGFR signaling can significantly reverse endocrine resistance.

Amplification of the fibroblast growth factor receptor 1 (FGFR1) is found in over 20% of ER^+^ mBC and is associated with resistance to endocrine therapy and CDK4/6 inhibitors.^146^^,^^147^ The combination of FGFR inhibitors and fulvestrant inhibits the growth of ER^+^/FGFR1-amplified tumors,^148^ and clinical studies combining various FGFR inhibitors (such as dovitinib, erdafitinib, futibatinib, and rogaratinib) and fulvestrant with or without palbociclib in ER^+^ mBC are showing an initial response rate (NCT01528345, NCT03238196, NCT04024436, and NCT04483505).^149^ Moreover, there is a clear crosstalk between GPER and FGFR1 activation. GPER mediates the non-classic estrogen pathway in the production of fibroblast growth factor 2 (FGF2)^150^^,^^151^ and promotes forward FGF2/FGFR1 paracrine activation coupling CAFs to BC cells for tumor progression.^152^ Therefore, the potential integration of GPER/FGFR-targeted agents should be further investigated to improve the outcome of HR^+^ mBC patients.

Activation of type I insulin-like growth factor receptor (IGF-IR) signaling promotes growth, metastasis, and drug resistance in BC.^153^ The combination of the IGF-IR inhibitor linsitinib and fulvestrant is more effective than the single agent in HR^+^ BC cells,^154^ and treatment with the IGF-IR monoclonal antibody is capable of overcoming resistance to hormone deprivation or anti-estrogen therapy.^155^ However, clinical trials of the IGF-IR inhibitor show disappointing results in solid tumors,^155^ indicating that the IGF-IR inhibitor alone is insufficient. Interestingly, insulin-like growth factor-I (IGF-I)/IGF-IR signaling up-regulates GPER expression and function in MCF-7 cells and CAFs, further participating in tamoxifen resistance.^156^, ^157^, ^158^, ^159^ More studies are needed to understand the poor sensitivity to the IGF-IR inhibitors and provide future strategies for the combined development of targeting GPER and IGF-IR inhibitors.

## GPER and non-genomic signaling activation

As previously described, multiple non-genomic signaling pathways elicited by GPER, such as the EGFR/MAPK/ERK and EGFR/PI3K/AKT pathways, are significantly involved in endocrine resistance in HR^+^ BC. We further confirm that a novel GPER-mediated cAMP/PKA/cAMP-response element binding protein (CREB) signaling triggers the energy metabolism reprogramming (EMR) in tumors, and that targeting cytoplasmic GPER in CAFs significantly reverses multiple drug resistance, including tamoxifen therapy, Her-2-targeted therapy, and chemotherapy.^160^ Simultaneously, acquired endocrine resistance in HR^+^ BC is accompanied by an intense GPER-induced intracellular Ca^2+^ mobilization,^72^^,^^93^ and estrogen regulates the Hippo signaling via the GPER/PLC/PKC pathway to enhance breast tumorigenesis.^161^ Whether the GPER-induced Ca^2+^ flux and PLC/PKC signaling directly induce endocrine resistance remains unreported.

## GPER and endocrine resistance gene alterations

GPER primarily elicits downstream gene transcription via non-genomic signaling. Connective tissue growth factor (*CTGF*), *EGR-1*, *C-FOS*, and *C-MYC* genes are identified as the target genes of GPER, and play a crucial role in participating in tamoxifen resistance of BC.^67^^,^^75^^,^^90^^,^^142^^,^^157^ Additionally, cyclins induced by GPER, such as cyclin A, cyclin D1, and cyclin E, exhibit critical CDK4/6 inhibitor resistance mechanisms involving altered activity of the CDK4/6-cyclin D complex, overexpression of the cyclin E-CDK2 complex, and CDK4/6 amplification.^102^^,^^107^^,^^108^ Our team has also determined that GPER promotes tamoxifen-resistance in ER^+^ BC cells by reducing B-cell lymphoma 2-like 11 (BCL2L11) proteins^162^ and mediates secondary drug resistance by regulating ABCG2 expression and membrane localization in tamoxifen-resistant BC.^74^ Therefore, the exploration of GPER-induced alterations in endocrine resistance genes will facilitate a better understanding of the underlying mechanism.

## GPER and ncRNA alterations

Increasing empirical data suggest that ncRNAs (such as short and long ncRNAs) are inextricably involved in a wide range of pathological processes including endocrine resistance.^163^ Among these short ncRNAs, multiple microRNAs are regulated by GPER in promoting tumor progression and endocrine resistance, such as miR-338-3p,^164^ miR-21,^165^ miR-144,^166^ miR-42,^167^ and miR-124.^168^ GPER-induced HOTAIR/miR-148a may enhance the ER axis and trigger tamoxifen resistance in HR ^+^ BC.^150^^,^^169^ We also reported a novel mechanism of GPER-induced lncRNA-Glu in regulating tumor glutamate secretion to increase cell invasion.^170^ Still, further clinical investigations are necessary to validate the correlation of GPER-induced ncRNAs with endocrine resistance.

## Mechanisms underlying GPER-mediated epigenetic and non-genetic variations

**Figure 5:**
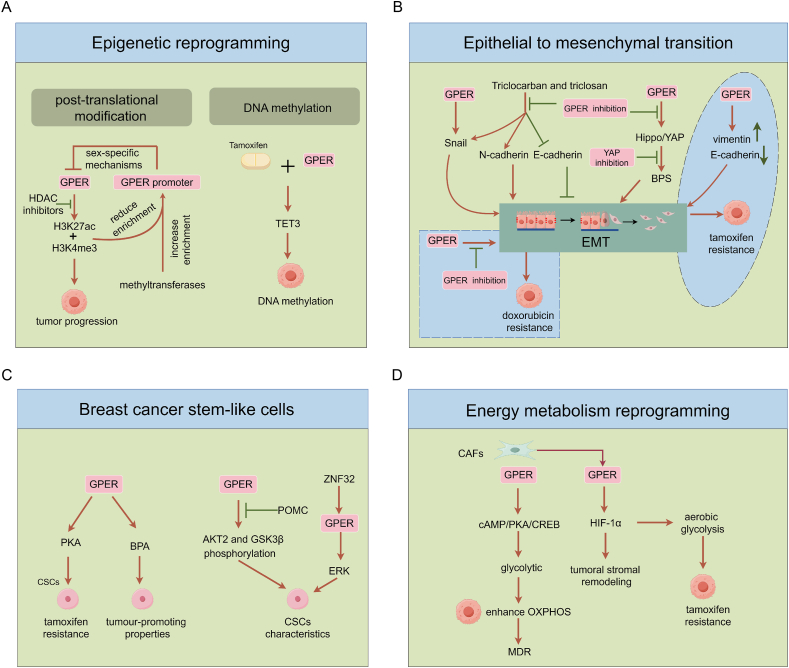
Mechanisms of GPER-mediated epigenetic and non-genetic variations. The roles of GPER in **(A)** tumor epigenetic reprogramming, **(B)** EMT, **(C)** breast CSCs, and **(D)** EMR are shown. GPER, G protein-coupled estrogen receptor; EMT, epithelial to mesenchymal transition; CSCs, cancer stem-like cells; EMR, energy metabolism reprogramming.

In numerous studies,^171^, ^172^, ^173^ associations between epigenetic and non-genetic variations among BC subtypes, resistance to endocrine therapy, or metastases have been established, and GPER is extensively shown to be implicated in these variations (Fig. 5). Here, we summarize the observations that support the major role of GPER-mediated epigenetic and non-genetic variations in endocrine resistance and emphasize the significance of a better understanding of their mechanisms.

## GPER and epigenetic reprogramming

Aberrant epigenetic regulations of estrogen signaling modulate chromatin accessibility in two ways, namely, i) through post-translational modification of chromatin-bound histones by histone acetyltransferases, histone deacetylases (HDAC), and histone methyltransferases, and ii) via DNA methylation conducted by DNA methyltransferases.^174^ In fact, epigenetic modifications mediated by GPER are closely examined in endocrine resistance events. On one hand, GPER governs epigenetic histone alterations (H3K4me3 and H3K27ac) to affect tumor progression,^175^^,^^176^ and sex-specific mechanisms are of utmost significance as the down-regulation of GPER is associated with promoter hypermethylation and reduced enrichment of H3K4me3 and H3K27ac marks around the GPER promoter.^177^ On the other hand, tamoxifen influences GPER-mediated DNA methylation patterns, particularly through modulating the expression mode of demethylase TET3.^178^ The potential of targeting GPER-mediated epigenetic modifications combined with HDAC inhibitors for individualized endocrine therapy provides a novel approach to improve treatment outcomes.

## GPER and epithelial to mesenchymal transition (EMT)

Resistance to endocrine therapies confers tumor cells an enhanced capacity for survival and metastasis, simultaneously accompanied by the manifestation of the EMT phenotype.^179^ A considerable number of studies have probed the GPER-mediated EMT phenomenon. We hypothesized that the acquisition of EMT constitutes a GPER-dependent novel pattern in tamoxifen-resistant BC cells.^72^^,^^73^ Mechanistically, several EMT transcription factors mediated by GPER (*e.g.*, Snail and Twist) and EMT markers (*e.g.*, N-cadherin, fibronectin, and vimentin) are involved. The activated GPER significantly increases the expression and nuclear localization of Snail and other key transcription factors of EMT.^180^^,^^181^ Triclocarban and triclosan down-regulate the expression of the epithelial marker E-cadherin but up-regulate the mesenchymal markers Snail and N-cadherin, a phenomenon that can be reversed by GPER inhibition.^182^ This inhibition reduces BC cell migration^182^ and emphasizes the role of GPER in EMT and metastasis development.^183^ In triple-negative BC, GPER/Hippo/YAP signaling is associated with bisphenol S-induced cell migration, and the inhibition of GPER/YAP axis blocks the bisphenol S-triggered cell migration and the up-regulation of fibronectin and vimentin.^184^^,^^185^ More notably, GPER up-regulates vimentin and down-regulates E-cadherin in tamoxifen-resistant MCF-7 cells and is also implicated in interleukin (IL)-6-induced migration, invasion, and tamoxifen resistance.^186^ The inhibition of GPER also sensitizes epithelial BC cells to doxorubicin by preventing EMT.^187^ In addition, beyond BC drug resistance, GPER-mediated EMT has been identified in other cancer subtypes, including gastric cancer,^188^ lung cancer,^189^ and cervical cancer.^190^ Thus, the available evidence suggests that GPER-mediated EMT is a prevalent clinical phenomenon in tumorigenic cells, highlighting its role in the potential hallmarks of BC and feasible therapeutic options to overcome endocrine resistance.

## GPER and breast cancer stem-like cells (CSCs)

Breast CSCs have been involved in endocrine resistance, and tamoxifen-induced CSC attributes are associated with the acquisition of tamoxifen resistance.^191^ Clinically, a significantly positive interrelationship between GPER and CD133 (a putative CSC marker) expression is discerned,^192^ and GPER/PKA signaling is essential for the survival of breast CSCs and potential tamoxifen resistance.^193^ These results can be further substantiated by other studies. For instance, physiological levels of BPA functionality in GPER-mediated CSCs underlie its remarkable tumor-promoting properties.^194^ Pro-opiomelanocortin is closely related to protein phosphorylation mediated by GPER in breast CSCs.^195^ Zinc finger protein 32 (ZNF32) participates in the GPER/ERK signaling and confers breast CSC characteristics, which may indicate an unfavorable prognosis for BC patients.^196^ Yet, the regulatory mechanisms between GPER and CSCs in endocrine resistance remain ambiguous, and more investigations are needed.

## GPER and tumor EMR

HR^+^ BC has been evidently demonstrated to evade endocrine therapy by transitioning to aberrant tumor EMR. Recent studies have indicated that endocrine and palbociclib-resistant cells show an increased dependency on oxidative phosphorylation and elevated levels of reactive oxygen species, thereby suggesting that oxidative phosphorylation serves as a prospective target for endocrine treatment-resistant HR^+^ BC patients.^197^ We determined that cytoplasmic GPER translocation in CAFs mediates the cAMP/PKA/CREB/glycolytic axis to endow tumor cells with enhanced oxidative phosphorylation through energy metabolism coupling, which further induces multiple drug resistance, including endocrine resistance.^160^ Clinical data have revealed that a high expression of cytoplasmic GPER in stromal fibroblasts predicts an unfavorable prognosis and a poor response to drug treatment.^160^ Other research teams have found that the GPER/hypoxia-inducible factor-1 alpha (HIF-1α, a key tumor EMR factor) axis acts as a principal regulator of peri-tumoral stromal remodeling in the fibrovascular tumor microenvironment,^198^ and GPER-mediated stabilization of HIF-1α contributes to the up-regulation of aerobic glycolysis in tamoxifen-resistant cells.^199^ In conclusion, GPER is directly involved in tumor EMR and mediates endocrine resistance, and targeting the GPER/EMR pathway may be one of the approaches to reverse resistant events.

## Mechanisms underlying GPER-aroused changes within the tumor microenvironment

**Figure 6:**
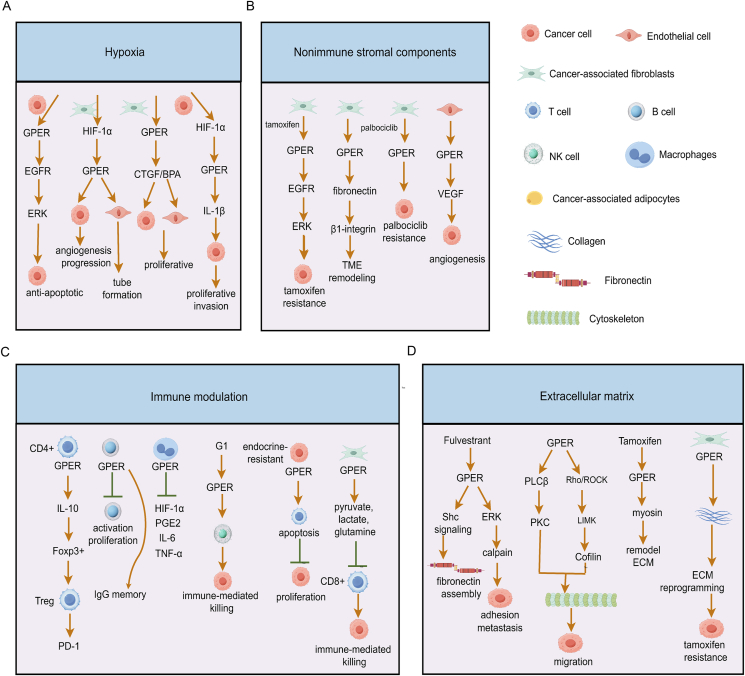
Mechanisms of GPER-aroused tumor microenvironment changes. **(A)** The functions of GPER under hypoxic conditions, and **(B)** the roles of GPER in non-immune stromal components (B), immune regulation (C), and ECM remodeling (D) are shown. GPER, G protein-coupled estrogen receptor; ECM, extracellular matrix.

There are intricate bidirectional signaling and reciprocal interactions between BC cells and the tumor microenvironment, which are significantly related to the biological behaviors of tumors, such as survival and metastasis.^200^ Several components of the tumor microenvironment, including hypoxia, non-immune stromal components, immune modulation, and the extracellular matrix (ECM), have been involved in endocrine resistance (Fig. 6).

## GPER and hypoxia

Hypoxia constitutes a crucial microenvironmental condition that distinguishes tumor from normal tissue and is associated with an unsatisfactory response to endocrine therapy.^173^ There is compelling evidence suggesting that GPER is closely interrelated with hypoxia and causes tamoxifen resistance.^199^ Mechanistically, hypoxia up-regulates GPER expression to exert anti-apoptotic effects via EGFR/ERK signaling in BC cells.^201^ HIF-1α/GPER signaling further triggers the expression of vascular endothelial growth factor (VEGF) in CAFs to enhance tumor angiogenesis and progression,^202^ and both HIF-1α and GPER are indispensable for VEGF-induced human vascular endothelial cell tube formation.^203^^,^^204^ Furthermore, GPER in CAFs governs hypoxia-driven BC invasion in a CTGF-dependent manner^205^ and BPA induces proliferative effects on both BC cells and vascular endothelial cells through a GPER-dependent pathway under hypoxia.^206^ Hypoxia initiates a functional association among HIF-1α, GPER, and the IL-1β/IL1R1 signaling towards a metastatic gene signature and a feed-forward loop of IL-1β that leads to BC proliferation and invasion.^207^ However, no studies have reported the involvement of GPER and hypoxia in the resistance to SERDs, CDK4/6 inhibitors, and other endocrine-targeted drugs.

## GPER and non-immune stromal components

CAFs are one of the principal cellular components within the breast tumor stroma and lead to angiogenesis, ECM remodeling, EMT, CSCs, and EMR by secreting growth factors, cytokines, proteases, and metabolites, thereby further facilitating tumor cell proliferation, invasion, metastasis, and clinical drug resistance.^200^ In fact, GPER is extensively involved in the aforementioned malignant biological behaviors. Specifically, our previous studies demonstrate that over 60% of breast tumor stromal fibroblasts express GPER,^160^ and other groups further show elevated GPER expression in CAFs from tamoxifen-resistant tumors compared with their sensitive counterparts, accounting for CAF proliferation and migration.^91^^,^^208^ In addition, tamoxifen up-regulates high mobility group box 1 (HMGB1) expression and secretion in CAFs via GPER/PI3K/AKT signaling to induce autophagy during the process of tamoxifen resistance.^208^ Other mechanisms of endocrine resistance include GPER in CAFs, enhancing fibronectin/β1-integrin signaling and lactate release to modify the tumor microenvironment conditions.^73^^,^^160^ The latest research shows that palbociclib induces pro-inflammatory transcriptional events via GPER signaling in CAFs, and microenvironmental GPER contributes to the reduced sensitivity to CDK4/6 inhibitor.^106^ This data suggests that GPER from CAFs plays a significant role in the crosstalk between the parenchymal and stromal components of endocrine-resistant BC. In recent years, the heterogeneity of breast CAFs has received increasing attention. Based on gene expression patterns, CAFs are predicted to perform multiple roles, such as inflammatory CAFs, myofibroblastic CAFs, matrix-producing CAFs, and vessel-associated CAFs,^209^^,^^210^ which might exert distinct effects in endocrine resistance. As the most prominent subgroup of CAFs (over 60%), the heterogeneity of GPER^+^ CAFs will be gradually explored in the future.

Tumor recurrence, metastasis, and clinical drug resistance caused by neovascularization constitute the main cause of mortality in BC patients, where vascular endothelial cells can reactivate dormant tumor cells.^211^ From the perspectives of tumor cells and CAFs, GPER-induced endothelial growth factor (VEGF) promotes angiogenesis and tumor progression by enhancing vascular endothelial tube formation.^143^^,^^202^, ^203^, ^204^, ^205^^,^^212^ GPER is involved in the responses upon endothelin-1 (ET-1) exposure, such as the migratory behavior of breast tumor cells and the formation of tube-like structures in human umbilical vein endothelial cells.^213^ Regarding vascular endothelial cells themselves, GPER contributes to the proliferation and migration of breast tumor-derived endothelial cells,^54^ and VEGF is up-regulated in a GPER-dependent manner by the treatment of BPA under hypoxic conditions in vascular endothelial cells.^206^ It seems that targeted angiogenesis might be effective in reversing endocrine resistance. However, in a phase II study of apatinib (an RTK inhibitor targeting VEGFR) plus exemestane in HR^+^ mBC (having progressed after previous letrozole or anastrozole treatment), the overall median progression-free survival is only 5.6 months, and patients with 0–1 line of chemotherapy for mBC showed no significant increase in median progression-free survival compared with those with more than 2 lines of chemotherapy (6.4 *vs*. 4.8 months; *p* = 0.09).^214^ This indicates that endocrine therapy in combination with VEGFR-targeted drugs alone may not be sufficient, and other combinatorial treatments such as targeting GPER are worthy of investigation.

Adipocytes, which are abundantly present in fat-rich breast tissue, are regarded as an actively functioning tissue involved in the secretion of various hormones and adipokines and the regulation of fuel availability.^215^^,^^216^ Obesity is correlated with the clinical response to HR^+^ BC, and peri-tumor adipocytes are gradually transformed by tumor cells to become cancer-associated adipocytes.^217^^,^^218^ In ovariectomized postmenopausal obese mice, activation of GPER leads to a reduction in adiposity and promotes the expression of genes associated with mitochondrial biogenesis and fatty acid oxidation in fuel utilization.^219^ The treatment of 3T3-L1 preadipocytes with the estrogen/GPER axis during differentiation inhibits lipid accumulation in adipocytes by disrupting mitotic clonal expansion.^220^ Thus, it is reasonably hypothesized that multiple functions of GPER-activated adipocytes are involved in the endocrine resistance of BC. Deletion of GPER triggers the activation of mitochondrial uncoupling respiration to induce adipose thermogenesis,^221^ and low-dose BPA regulates inflammatory cytokines through GPER in mammary adipose cells.^222^ Recently, direct evidence shows that cancer-associated adipocytes are found to induce tamoxifen resistance by activating the PI3K/AKT/mTOR pathway in HR^+^/GPER ^+^ BC,^191^ thereby making it necessary that more translational research efforts should be focused on this area.

## GPER and tumor immune regulation

Tumor immune regulation plays a cardinal role in proliferation, invasion, and metastasis, and exerts an influence on the therapeutic susceptibility and prognosis of malignancies.^223^ The interaction between GPER and immune cells is widely manifested. GPER is expressed in T cells, B cells, monocytes, and macrophages, exerting immune effects and shaping the immune microenvironment.^224^ GPER influences thymic functionality and triggers the expression of IL-10 in CD4^+^ T cells,^225^ up-regulates forkhead box protein expression, and positively regulates programmed cell death-1 (PD-1) in T regulatory cells.^226^ GPER reduces activation-induced proliferation of B lymphocytes and facilitates IgG memory.^227^ In monocytes/macrophages, GPER decreases the expression of Toll-like receptor 4 (TLR4), and inhibits the expression of prostaglandin E2, IL-6, and tumor necrosis factor-α (TNF-α).^228^ The GPER-specific agonist G1 enhances immune-mediated killing independently of ER status and increases susceptibility to natural killer cell death.^229^

In endocrine-resistant HR^+^ mBC, GPER induces T-lymphocyte-related apoptosis, resulting in the inactivation of the majority of immune cells infiltrating breast stroma and influencing tumor proliferation.^230^^,^^231^ Tamoxifen resistance-induced extracellular vesicles reduce immune cell activation and the expression of immune checkpoint molecules.^232^ Fulvestrant and BPA also predispose an immunosuppressive tumor microenvironment via GPER,^229^^,^^233^ further suggesting that GPER may achieve tumor immune evasion via endocrine resistance in HR^+^ mBC. More convincingly, we discovered that GPER-induced metabolites (pyruvate, lactate, and glutamine) from CAFs also compromised the immune function of CD8^+^ T cells, leading to BC immune escape (unpublished data). Thus, recapitulating the immunoregulatory mechanism of endocrine-resistant tumors provides insights into the potential clinical significance of combining targeting GPER with immunotherapy in HR^+^ mBC.

## GPER and ECM remodeling

ECM is responsible for modulating the proliferation, migration, and clinical drug susceptibility of surrounding cells, and it is composed of collagen, fibronectin, laminin, and elastin.^234^ Bioinformatics analysis indicated that GPER was associated with pro-migration and pro-metastasis signaling pathways, such as cell adhesion molecules, ECM-receptor interaction, and focal adhesion routes.^235^^,^^236^ Fulvestrant induces the coordination of GPER-mediated fibronectin matrix assembly and growth factor release through a Shc-dependent signaling^237^^,^^238^ and activates the GPER-mediated ERK/calpain/cell adhesion pathway to enhance metastasis and reduce the efficacy of antiestrogens in the treatment of HR^+^ BC.^239^ GPER also mediates cytoskeleton assembly and facilitates BC cell migration via the PLCβ-PKC and Rho/ROCK-LIMK-Cofilin axis.^240^ Tamoxifen via GPER affects myosin-dependent contractility and matrix stiffness mechanosensing to remodel ECM.^241^ Interestingly, GPER-inducing cellular cytoskeletal remodeling can also be observed in pan-tumors, such as testicular germ cell tumor,^242^ ovarian cancer,^243^ CAFs,^244^ and T-cell lymphoblastic leukemia.^245^ GPER expression could also influence the expression of collagen-1 (COL1A1) in CAFs,^246^ and we hypothesized that GPER-mediated ECM reprogramming could promote tamoxifen resistance by strengthening communication with the tumor microenvironment via the fibronectin/β1-integrin pathway.^73^ Collectively, these studies suggest that targeting ECM components in combination with GPER and ER might provide substantial clinical benefits in endocrine-resistant tumors.

## Future directions and clinical perspective for GPER

Although the roles and mechanisms of GPER in endocrine resistance of HR^+^ BC have been studied from multiple perspectives, its clinical transformation still requires in-depth exploration at least in the following domains: i) It is essential to integrate precise and effective gene detection techniques (such as whole genome sequencing^247^ and single-cell RNA sequencing^248^) to investigate the activation of GPER signaling and its downstream clinical markers in drug-resistant tumors, further guiding individualized clinical treatment and prognosis determination in HR^+^ mBC with higher accuracy; ii) Circulating tumor cells are regarded as one of the fundamental causes of relapse in BC,^249^ and therefore the biological functions of GPER and circulating tumor DNA in these cells still need investigation; iii) The organ-specific niche plays a crucial role in tumor organotropic metastasis. Recently, our team confirmed that tryptophan 2,3-dioxygenase (TDO2)-positive matrix fibroblasts facilitate lung-specific metastasis and immune evasion of disseminated tumor cells in the metastatic niche.^250^ Given that HR ^+^ mBC is highly prone to metastasize to bone compared with other types,^251^ GPER could play a role in this bone-specific metastasis after resistance to endocrine therapy. iv) The clinical utilization of GPER may facilitate better classification of luminal type BC. It is widely known that over 70% of BC with ER^+^ and approximately 70% of tumor tissues express GPER in ER^+^ patients. Thus, we can further categorize these HR^+^ patients into four subtypes based on GPER and HER2 status (Table 3). The ER^+^/GPER^+^/HER2^−^ subtype (about 60% in HR^+^ BC) requires dual inhibition of both ER and GPER. The new triple-positive subtype (ER^+^/GPER^+^/HER2^+^, accounting for approximately 10%) demands more targeted inhibition of ER, GPER, and HER2. Similarly, conventional dual-targeted therapy to ER and HER2 is sufficient for the ER^+^/GPER^−^/HER2^+^ subtype, which constitutes only 5%, indicating that two-thirds of classical triple-positive (ER^+^/PR^+^/HER2^+^) BC treatments still need further improvement because of the existence of GPER. Classical endocrine therapy alone is only sufficient for the ER^+^/GPER^−^/HER2^−^ subtype (accounting for 25%). However, this classification method requires more clinical validation.

**Table 3:** Clinical classification of HR^+^ BC. HR^+^ BC can be further classified based on the expression of GPER and HER2, which are associated with the prognosis and the indicated clinical treatment approaches. BC, breast cancer; HER2, human epidermal growth factor receptor 2; HR^+^, hormone receptor-positive; GPER, G protein-coupled estrogen receptor.

HR^+^ subtypes	ER^+^/GPER^+^/HER2^-^	ER^+^/GPER^+^/HER2^+^ (New triple-positive)	ER^+^/GPER^−^/HER2^+^ (Classical triple-positive)	ER^+^/GPER^−^/HER2^-^
Proportion	60%	10%	5%	25%
ER	+	+	+	+
PR	+/−	+/−	+/−	+/−
GPER	+	+	–	–
HER2	–	+	+	–
Possible prognosis	Intermediate	Poor	Intermediate	Good
Potential treatment	Endocrine therapy and GPER-targeted therapy	Endocrine therapy, GPER-targeted and HER2-targeted therapy	Endocrine therapy and HER2-targeted therapy	Endocrine therapy

Another major focus of GPER-related research should be on preventing or overcoming resistance to the combination of endocrine therapy and CDK4/6 inhibitors, as CDK4/6 inhibitors currently are a standard treatment for HR^+^ early BC and mBC. The resistance mechanisms of CDK4/6 inhibitors involve numerous pathways, such as genetic, morphological, and clinical heterogeneity,^252^ and have substantial crosstalk with GPER signaling. For instance, a PIK3CA-mutated HR^+^/GPER^+^ mBC may imply that PAM inhibitors should be used in combination with GPER-targeted drugs. In HR^+^ mBC, the clinical options after first-line treatment with CDK4/6 inhibitors remain controversial.^253^ Furthermore, aside from PAM inhibitors, other CDK4/6 inhibitors re-challenge,^254^ and oral SERDs,^255^ HDAC inhibitors,^256^ chemotherapy,^257^ and HER2-or trophoblast cell surface antigen 2-targeted antibody conjugating drugs^258^^,^^259^ have been shown to improve progression-free survival or overall survival in these patients, making these options available as clinical alternatives. In the future, it is feasible to identify the optimal second-line treatments through targeted therapy combined with GPER.

Drug development for GPER and the implementation of corresponding clinical studies targeting GPER in endocrine-resistant tumors will constitute the core clinical task in the coming years. Despite the existence of numerous small-molecule approaches that indicate targeting inhibition of GPER both *in vitro* and *in vivo* (Table 4),^260^, ^261^, ^262^, ^263^, ^264^, ^265^, ^266^, ^267^, ^268^, ^269^, ^270^, ^271^, ^272^, ^273^, ^274^, ^275^ the therapeutic efficacy of these compounds has yet to be verified in the clinical setting. Interestingly, targeting GPER emerges as an outstanding ally for immunotherapy, including anti-PD-1, anti-programmed cell death-ligand 1 (anti-PD-L1), and anti-cytotoxic T lymphocyte-associated antigen 4 (anti-CTLA4) therapeutics. By leveraging a public dataset for predicting biomarkers of immunotherapy effects in pan-tumors,^276^ a sex-specific phenomenon is observed that elevated GPER expression reveals a markedly suboptimal immunotherapy response in female patients (Fig. 3E–G) relative to that of male patients (Fig. 3H–J). The recently developed LNS8801, an orally highly selective small molecule GPER agonist, normalizes the level of c-Myc in several cancer cells (excluding BC), inhibits tumor proliferation and invasion, and enhances immune recognition.^277^, ^278^, ^279^, ^280^, ^281^ The combinatorial treatment of LNS8801 and pembrolizumab (a PD-L1 inhibitor) has entered phase 1/2 clinical trials (NCT04130516), which represents the first clinical study of GPER in several solid tumors (excluding BC). Preliminary data suggest that the combination therapy shows encouraging anti-tumor activity in patients with pembrolizumab resistance and is well tolerated.^282^ Another phase 2/3 clinical trial of LNS8801 with or without pembrolizumab in patients with refractory melanoma is scheduled to start in November 2024 (NCT06624644). Interestingly, GPER assumes immunosuppressive functions in BC,^229^, ^230^, ^231^, ^232^, ^233^^,^^283^, ^284^, ^285^ which contrasts sharply with its immunoactivating roles in other solid tumors like melanoma, pancreatic, gastric, lung, and colorectal cancers.^286^ Therefore, GPER may have potential as a screening biomarker for drug sensitivity in the clinical setting. Developing a highly selective GPER-antagonist drug for use alone or in combination with other therapeutic modalities may improve the prognosis of HR^+^ BC.

**Table 4:** Summary of small molecule approaches targeting inhibition of GPER in BC. BC, breast cancer; GPER, G protein-coupled estrogen receptor.

GPER-targeting inhibition drugs	Mechanism	Concentration used	Research phase	Reference
G15	GPER-specific antagonist	*In vitro*: 10nM-10μM *In vivo*: 40μg/kg-1.46 mg/kg	Preclinical research	^[^72, 73, 74^,^^92^^,^^109^^,^^163^^,^^166^^,^^187^^,^^267^, ^268^, ^269^, ^270^^]^
G36	GPER-specific antagonist	*In vitro*: 50nM-1μM *In vivo*: 50 μg/kg	Preclinical research	^[^271, 272, 273^]^
MIBE (ethyl 3-[5-(2-ethoxycarbonyl-1-methylvinyloxy)-1-methyl-1H-indol-3-yl]but-2-enoate)	GPER antagonist	*In vitro*: 10μM-10mM	Preclinical research	^[^^268^^]^
CIMBA (2-cyclohexyl-4-isopropyl-N-(4-methoxybenzyl)aniline)	GPER antagonist	*In vitro*: 100nM-10μM *In vivo*: 16–32 μg/kg	Preclinical research	^[^^274^^]^
Endocrine-disrupting chemicals (six kinds)	GPER antagonist	*In vitro*: 4-Tert-Octylphenol: 100 nM Fenitrothion: 1 μM Cypermethrin: 1 μM Azoxystrobin: 1 μM Malathion: 10 nM Deltamethrin: 1 μM	Preclinical research	^[^^275^^]^
Estriol	GPER antagonist	*In vitro*: 100nM-1μM	Preclinical research	^[^^276^^]^
Benzopyrroloxazine (two kinds)	GPER antagonist	*In vitro*: PBX-1: 10 μM PBX-2: 10 μM	Preclinical research	^[^^277^^]^
C4PY (meso-octamethylcalix[4]pyrrole)	GPER antagonist	*In vitro*: 1 μM	Preclinical research	^[^^278^^]^
Pyrrolo[1,2-a]quinoxaline 14 c	GPER antagonist	*In vitro*: 1 μM	Preclinical research	^[^^279^^,^^280^^]^
ERα17p	GPER inverse agonist	*In vitro*: 10 μM *In vivo*: 2 mg/kg	Preclinical research	^[^^281^^,^^282^^]^
HO-AAVPA (N-(2-Hydroxyphenyl)-2-Propylpentanamide)	Decreasing GPER expression	*In vitro*: 0.142mM–0.283 mM	Preclinical research	^[^^283^^]^
GPER-targeting PROTACs (UI-EP001 and UI-EP002)	GPER-targeted degradation	*In vitro*: 10 μM	Preclinical research	^[^^101^^]^

## Conclusions

**Figure 7:**
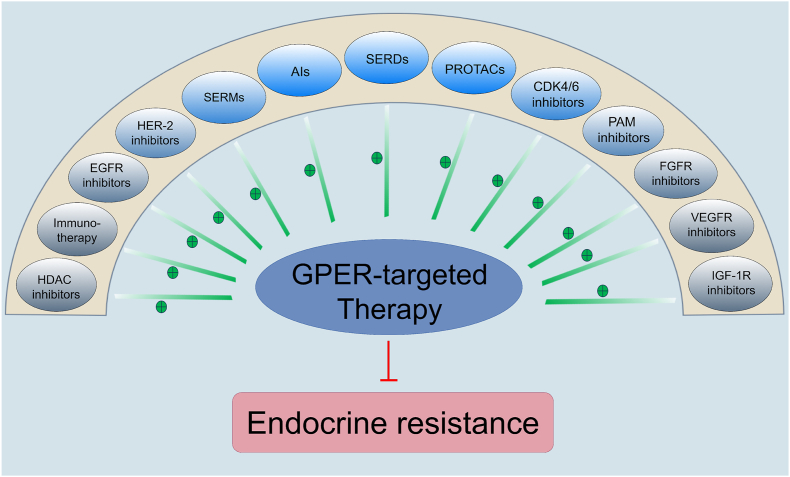
Potential therapeutic strategies combined with GPER-targeted therapy to combat endocrine resistance in the clinic. GPER, G protein-coupled estrogen receptor.

Although the majority of HR^+^ early BC is susceptible to conventional endocrine therapeutics, the occurrences of endocrine resistance still present an obstacle to the cure of HR^+^ mBC. Given that GPER constitutes approximately up to 70% of HR^+^ BC and is involved in endocrine resistance through multiple mechanisms, such as somatic mutations, epigenetic and non-genetic variations, and changes within the tumor microenvironment, GPER is regarded as an optimal target for reversing endocrine resistance (Fig. 7). Thus, GPER-targeted combination therapeutics for high-risk HR^+^ early BC and dormant BC cells can be employed to achieve the maximum radical cure and prevent recurrence. Further studies are needed to translate these discoveries into more effective clinical approaches.

## CRediT authorship contribution statement

**Tenghua Yu:** Writing – original draft, Supervision, Funding acquisition, Formal analysis, Data curation, Conceptualization. **Chongwu He:** Writing – original draft, Software, Funding acquisition, Conceptualization. **Hui Zhang:** Data curation. **Yi Zhu:** Data curation. **Annie Wang:** Writing – review & editing. **Xiaoqiang Zeng:** Software, Data curation. **Yanxiao Huang:** Software, Data curation. **Jiamin Zhong:** Data curation. **Xingye Wu:** Data curation. **Yi Shu:** Data curation. **Guowei Shen:** Data curation. **Chao Yu:** Data curation. **Ke Zhou:** Formal analysis, Data curation. **Usman Zeb:** Formal analysis, Data curation. **Rebeka Dejenie:** Data curation. **Yan Peng:** Data curation. **Rex C. Haydon:** Writing – review & editing. **Hue H. Luu:** Writing – review & editing. **Russell R. Reid:** Writing – review & editing, Funding acquisition. **Tong-Chuan He:** Writing – review & editing, Funding acquisition, Data curation, Conceptualization. **Jiaming Fan:** Data curation. **Jingjing Li:** Writing – original draft, Supervision, Data curation, Conceptualization.

## Funding

This work was supported in part by research grants from the 10.13039/501100001809National Natural Science Foundation of China (No. 82160565, 82260565, 82104289), the Jiangxi Province Ganpo Talent Support Program (China) (No. 20232BCJ23035), the Youths Program of the 10.13039/501100004479Natural Science Foundation of Jiangxi Province, China (No. 20212BAB216063), the Science and Technology Research Project of 10.13039/501100009102Jiangxi Provincial Department of Education (China) (No. GJJ2203530), the Research Open Fund Project of Jiangxi Cancer Hospital (China) (No. KFJJ2023ZD01, KFJJ2023YB06), the Shandong Provincial Health Commission of China (No. M-2022053), the Science and Technology Innovation Plan from 10.13039/501100010886Weifang Medical University (Shandong, China) (No. 041004), the Yuandu Scholar Grant of Weifang City, Weifang Science and Technology Bureau Plan Project (Shandong, China) (No. 2021YX081), the Science and technology project jointly established by the Science and Technology Department of the 10.13039/501100005891State Administration of Traditional Chinese Medicine (China) (No. GZY-KJS-SD-2023-079), the Shandong Provincial Medical Association Young Talent Promotion Project (China) (No. 2023_GJ_0039), the Science and Technology Innovation Plan from 10.13039/501100010886Weifang Medical University (Shandong, China) (No. 041011), and the 10.13039/100000002US National Institutes of Health (No. CA226303 to T.C.H.; DE030480 to R.R.R.). T.C.H. was supported by the Mabel Green Myers Research Endowment Fund and 10.13039/100007234The University of Chicago Orthopaedics Alumni Fund. Funding sources were not involved in the study design; in the collection, analysis, and interpretation of data; in the writing of the report; and in the decision to submit the paper for publication.

## Conflict of interests

T.C.H. is one of editors-in-chief for *Genes & Diseases,* and he was not involved in the editorial review or the decision to publish this article. The authors declare that they have no known competing financial interests or personal relationships that could have appeared to influence the work reported in this paper.
